# Measuring Cyber Interpersonal Violence in Adolescents: Development and Validation of the CyIVIA Instrument

**DOI:** 10.3390/ejihpe15110218

**Published:** 2025-10-25

**Authors:** Bárbara Machado, Isabel Araújo, Rui Ferreira Jesus, Estela Vilhena, Ricardo Castro, Paula Lobato de Faria, Sónia Caridade

**Affiliations:** 1 NOVA National School of Public Health, NOVA University of Lisboa, 1600-560 Lisboa, Portugal; 2 The Artificial Intelligence and Health Research Unit, Polytechnic University of Health, CESPU, 4760-409 Vila Nova de Famalicão, Portugal; isabel.araujo@ipsn.cespu.pt; 3 iHealth4Well-Being—Innovation in Health and Well-Being—Research Unit, Polytechnic University of Health, 4560-462 Penafiel, Portugal; rui.jesus@ipsn.cespu.pt; 4 2Ai—School of Technology, Polytechnic Institute of Cávado and Ave (IPCA), 4750-810 Barcelos, Portugal; evilhena@ipca.pt; 5 LASI—Associate Laboratory of Intelligent Systems, 4800-067 Guimarães, Portugal; 6 Higher School of Technology and Management, Polytechnic Institute of Viana do Castelo, Alto Minho, 4900-644 Viana do Castelo, Portugal; rapc@estg.ipvc.pt; 7 Interdisciplinary Centre for Social Sciences (CICS), Comprehensive Health Research Centre (CHRC), National School of Public Health, NOVA University, 1600-560 Lisboa, Portugal; pa.lobfaria@ensp.unl.pt; 8 Psychology Research Centre, School of Psychology, University of Minho, 4710-057 Braga, Portugal; scaridade@psi.uminho.pt

**Keywords:** adolescents, cyber violence, interpersonal violence, digital practices, public health

## Abstract

Background: The growing prevalence of cyber interpersonal violence (CIV) among adolescents necessitates tools to assess its dimensions: victimization, perpetration, and bystander roles. This study develops and validates the “Cyber Interpersonal Violence Instrument for Adolescents” (CyIVIA). Method: CyIVIA’s development involved 253 adolescents, comprising 146 boys and 107 girls, aged between 11 and 16 years, from northern Portugal. The 40-item Likert-scale tool assesses direct (victimization and perpetration) and indirect (bystander roles) CIV. Analyses included exploratory and confirmatory factor testing for reliability and validity. Results: CyIVIA demonstrated internal consistency (α = 0.743–0.851) and a robust multidimensional structure. 56.5% reported CIV involvement, with gender differences: girls showed higher victimization, and boys, higher perpetration. The 8th grade emerged as a key intervention period. Conclusions: CyIVIA is a reliable tool for assessing CIV among adolescents. Interventions should focus on prevention, empower active bystanders, and promote safer digital environments.

## 1. Introduction

The global threat of cyberviolence is rising (Machado et al., 2024), with increased online access exposing children and adolescents to more significant risks (De Coninck et al., 2023; Jun, 2020). Adolescents, particularly vulnerable, face more severe mental, social, and cultural impacts from online violence (Li et al., 2024; Winarti & Saudah, 2023).

Although there is no universally accepted definition of “cyberviolence,” the term is used widely across academic disciplines (Barker & Jurasz, 2024; Lian et al., 2022). Internationally, terms like cyberbullying, cyberaggression, online violence, and digital crime are employed (Barker & Jurasz, 2024). Existing definitions often fail to capture the full scope of emerging online violent behaviors. The rise of the “Born-Digital” generation, widespread access to smart devices, and the shift to online education during the COVID-19 pandemic have contributed to a decrease in the age of both victims and perpetrators of cyberviolence (Lian et al., 2022). As the phenomenon evolves, precise and updated terminology is essential for research and policy. Despite this difficulty, many authors have studied the phenomenon.

This crescent concerning cyberviolence has resulted in the development of measurement instruments with several characteristics, focusing on the main dimensions of Cyber Interpersonal Violence (CIV). CIV encompasses various forms of abuse, including psychological, physical, sexual, controlling behaviors, and direct aggression, like the types of abuse seen in face-to-face dating violence (Caridade & Braga, 2019) and comprises cyber dating abuse (CDA), cyberbullying and cyber-harassment. Cyberbullying is understood as a form of bullying conducted via electronic media (Dooley et al., 2009). Among children and adolescents, it involves intentional and repeated harm inflicted by one or more peers using computers, smartphones, and other digital devices, occurring within cyberspace (Jadambaa et al., 2019; Zhu et al., 2021). Parents play a crucial role in preventing and supporting their children against cyberbullying. However, for a deeper comprehension of the phenomenon, it is crucial to conduct more studies about the subject (Marinoni et al., 2024), namely, scales that allow us to understand cyberviolence in its various forms better.

From a Social–Ecological Theory perspective, understanding cyberbullying requires assessing the multiple roles adolescents may assume—cyberbully, cyber victim, cyberbully–victim, and noninvolved—within the broader context of individual, familial, peer, and school influences. Despite this complexity, most existing instruments focus on isolated roles, limiting the capacity to identify overlapping behaviors and associated risk factors. Developing a tool that evaluates all cyberbullying roles comprehensively is essential to reflect the multifactorial nature of the phenomenon and to inform targeted interventions that consider both shared and unique predictors across ecological levels (Guo et al., 2021).

Scoping reviews (Machado et al., 2022) have confirmed that although progress has been made in developing validated measures (Caridade & Braga, 2019; Buelga et al., 2020), the majority of them are only concerned about one specific area, and they need to be consistent with methodology and terminology (Geffner, 2016).

Assessment tools have been developed during recent years for all CIV issues. Although some tools have problems with the psychometric characteristics of the instruments, some have successfully overcome these instrument validation difficulties and developed good instruments to access CDA (Sánchez Jiménez et al., 2023; Redondo et al., 2022). The Cyberdating Q_A Scale (Sánchez et al., 2015) is one that was validated for the Spanish population and sought to analyse how teenagers use new technologies in their intimate relationships, considering six dimensions (OC: Control online; OJ: Online Jealousy; OIB: Intrusive Online Behaviour; OI: Online Intimacy; ECS: Emotional Communication Strategies; CP: Cyberabuse Practices). The Cyber Dating Violence Inventory (CDVI) (Morelli et al., 2018) has emerged, and evaluates verbal/emotional, relational, and threat violence and examines violence reciprocity from adolescence onward, showing good psychometric properties. To address limitations in existing measures, Borrajo et al. (2015) developed the Cyber Dating Abuse Questionnaire (CDAQ), which covers a broad range of abusive behaviours, offering a comprehensive view of cyber abuse in both victimization and perpetration contexts. This initial measure was later validated in other cultures, including Brazil (Zumalde et al., 2021), Portugal (Caridade & Braga, 2019), Mexico (Hldalgo-Rasmussen et al., 2020) and Chile (Lara, 2020).

Cyberbullying research faces challenges due to a need for more consensus on its definition and measurement (Baldry et al., 2016; Englander et al., 2017; Garett et al., 2016). However, this issue is addressed by instruments like the European Cyberbullying Intervention Project Questionnaire (Del Rey et al., 2015) and the Adolescent Cyber-Aggressor Scale (CYB-AGS) (Buelga et al., 2020). The former evaluates victimization and aggression, incorporating repetition and power imbalance. The CYB-AGS, an update of the CYB-AG scale (Buelga & Pons, 2012), includes 18 Likert-scale items assessing direct and indirect cyberbullying over 12 months.

Cyber harassment, a form of CIV, involves persistent digital aggression, such as invading privacy, sexual harassment, and monitoring (da Silva Pereira et al., 2015; Hazelwood & Koon-Magnin, 2013; Zweig et al., 2013). It often overlaps with cyber-stalking and obsessive behaviors (Spitzberg & Cupach, 2014). In Portugal, 33.1% of adolescents engage in cyber-harassment, and 93.3% are both aggressors and victims (Novo et al., 2014) and awareness campaigns remain infrequent (Pereira et al., 2016). Tools like the Cyber-harassment Assessment Scale evaluate perpetration and victimization but exclude bystander roles. Bystanders, critical in cyberviolence dynamics, mostly remain passive due to fear, skill deficits, or normalized violence (Machado et al., 2024; Sobol et al., 2024). Active bystanders, motivated by empathy and self-efficacy, can reduce the severity of incidents (Patterson et al., 2017). Females intervene more often, especially when the victim is a friend (Rebollo-Catalan & Mayor-Buzon, 2020). However, online anonymity weakens accountability, reinforcing negative behaviors (Jeyagobi et al., 2022).

Educational programs should foster empathy, build intervention skills, and address anonymity barriers to empower bystanders and promote safer online environments (Machado et al., 2024; Sobol et al., 2024).

### The Current Study

In Portugal, there is a need for a validated instrument to assess CIV across victim, aggressor, and bystander perspectives. Existing tools focus on isolated dimensions, lacking integration. A comprehensive tool can address this gap, support school programs, and raise awareness among adolescents and educators.

The present study aims to develop, validate and refine a new assessment measure for CIV among adolescents. Specifically, the aims of the present study are (1) to develop and validate a new instrument for assessing cyber interpersonal aggression, victimization and bystanders’ knowledge about CIV; (2) to test and refine this version of the instrument; (3) to determine the prevalence of CIV among adolescents.

To achieve the proposed goals, we conducted a literature review (Machado et al., 2022) to gain a deeper understanding of the significant concepts and design some of the preliminary instruments. Second, we conduct a qualitative project, with focus groups with adolescents to understand the phenomenon from their point of view (Machado et al., 2024). Moreover, we developed and validated an instrument that was applied to adolescents to measure CIV. At least, to estimate discriminant validity, we used the Brief Symptom Inventory (BSI), a widely validated self-report instrument that assesses psychological symptoms and distress (Canavarro et al., 2017; Nazaré et al., 2017).

## 2. Materials and Methods

The study included 253 adolescents (11–16 years, M = 12.6; SD = 1.19) from four public schools in northern Portugal. Boys comprised 55.7% (*n* = 146) and girls 42.3% (*n* = 107). Most were in the 6th (42.3%, *n* = 107) or 8th grade (36%, *n* = 91). Nearly all students had a computer at home (95.3%, *n* = 241). Internet use varied: 37.9% (*n* = 96) spent 3–4 h daily, 33.6% (*n* = 85) 1–2 h, 22.9% (*n* = 58) over 4 h, and 5.5% (*n* = 14) under 1 h.

The instrument was developed in 4 phases: (a) Literature review, (b) Focus groups with adolescents, (c) Review by experts, and (d) Creation of the final instrument. Based on the literature review and in the focus group, 56 items were initially developed and subsequently submitted to content analysis by a panel of experts, which resulted in the elimination of 16 items due to redundancy or lack of clarity. This analysis returned 40 behavioral items that were grouped into four dimensions: victimization (10 items), perpetration (10 items), bystander knowledge of perpetration (10 items) and bystander knowledge of victimization (10 items) (cf. Table 1).

Afterwards, following the pre-test, the remaining items were subjected to statistical item analysis (item-total correlations, factor loadings, and reliability indices), leading to the elimination of an additional 18 items. Thus, the final version of the instrument comprised 22 items, ensuring both content validity and internal validity (cf. Table 1).

(a) Literature review

We reviewed the extant literature on adolescent cyber interpersonal violence to identify the theoretical dimensions, the most representative behaviors of the construct, the principal limitations of previous studies and the main developed instruments in the field (Machado et al., 2022). We also revised the main instruments produced, which comprised CIV: Cyber Dating Abuse (CDA) (Borrajo et al., 2015; Caridade & Braga, 2019; Morelli et al., 2018; Smith et al., 2019), Cyberbullying (Buelga et al., 2020; Del Rey et al., 2015; Jun, 2020), and Cyber-harassment (Marret & Choo, 2017; Powell & Henry, 2018; Sánchez et al., 2015).

(b) Focus group with young adults

In phase two, 15 focus groups with 42 boys and 66 girls (11–15 years, M = 12.87; SD = 0.31) explored adolescents’ views on cyber interpersonal violence (CIV) to inform the final scale (Machado et al., 2024). Of the 15 focus groups, five included only girls, five only boys, and five were mixed. We ensured that at least one of the groups contained participants from all stages (6th to 9th grade). Notably, none of these participants completed the present instrument, despite attending the same schools.

Thematic analysis revealed multiple dimensions of CIV. Participants described forms of violence (emotional, physical, and sexual abuse) and typologies such as cyberbullying, cyber dating abuse, and cyberstalking. They also reported a range of abusive behaviors, including insults, rumour spreading, posting/sharing photos or videos without consent, hacking, image manipulation, threats, and repeated or escalating attacks.

Adolescents identified several factors associated with CIV. At the *individual level*, they pointed to impulsivity, the need for peer acceptance, anonymity, envy, discrimination (e.g., race, disability), mental health issues, low self-esteem, prior victimization experiences, sadness, and family education that normalized violence. At the *relational level*, jealousy and revenge were emphasized, while at the *family level*, divorce, domestic violence, bereavement, and economic hardship were considered facilitators. In addition, the ubiquity of digital practices, lack of parental control, and the speed and permanence of online content dissemination were seen as major facilitators of CIV.

Participants also discussed victim and aggressor profiles. Aggressors were often perceived as male, sometimes sharing vulnerabilities with victims (e.g., shyness), and occasionally using violence to feel superior. Victims were described as more often female and characterized by physical (e.g., wearing glasses, acne, disability), psychological (e.g., shyness, low self-esteem), sociodemographic (e.g., sexual orientation, ethnicity, migration status), or functional (e.g., socioeconomic disadvantage) vulnerabilities.

When reflecting on victims’ responses, adolescents reported both passive strategies (e.g., ignoring the abuse, remaining silent) and active strategies (e.g., reporting to parents, teachers, psychologists, peers, or police; confronting aggressors; leaving group chats; creating private accounts). Some highlighted the absence of help-seeking due to mistrust or fear of ridicule.

The role of bystanders also emerged strongly. Supportive bystanders encouraged victims to report, offered direct help (e.g., blocking aggressors), or sought adult intervention, while others ignored the incidents, minimized their importance, or disclosed them informally.

Regarding the perceived impacts of CIV, participants highlighted profound *emotional consequences* (sadness, shame, fear, anger, insecurity, feelings of inferiority, depression, loneliness, humiliation, anxiety) and *behavioral consequences* (self-harm, suicidal ideation or attempts, social isolation, disordered eating, and cyber revenge).

Finally, the theme of gender and CIV was salient. Adolescents discussed stereotypical gender roles (e.g., girls expected to play with dolls, boys expected to excel in sports) and societal values, such as rigid beauty standards applied to both genders—though perceived as particularly intense for girls. They also emphasized gender differences in impact: boys were viewed as less affected by insults or appearance-related jokes. At the same time, girls were considered more vulnerable, especially to appearance-based bullying and peer-to-peer aggressions.

Overall, participants identified public violence and multimedia content as the most severe forms and emphasized control and surveillance as the most common issues (Machado et al., 2024). These findings informed of the preliminary item pool for the scale, ensuring cultural specificity and contextual sensitivity (Machado et al., 2024).

(c) Review by experts

A questionnaire was developed based on the scoping review and focus group findings. A panel of three experts refined the items, improving linguistic clarity and organizing them into four dimensions:

1. Victimization (10 items): suffering from different forms of abusive behavior using technological devices.
2. Perpetration (10 items): perpetrating different forms of abusive behavior using technological devices.
3. Bystander knowledge of perpetration (10 items): Knowing someone who perpetrates different forms of abusive behavior using technological devices.
4. Bystander knowledge of victimization (10 items): Knowing someone who suffers from different forms of abusive behavior using technological devices.

All dimensions considered all three categories of CIV, such as so CDA, Cyberbullying and Cyber harassment.

(d) Creation of the final scale

The final Cyber Interpersonal Violence Instrument for Adolescents (CyIVIA) comprised two parts: one about direct CIV (comprises victimization and perpetration) and a second part about indirect CIV (bystander position), in a total of 40 items (20 + 20) rated on a 5-point Likert-type scale ranging from 1 = ‘This never happened to me’ to 5 = ‘This always happened to me’, which measures the frequency with which respondents have experienced the behaviour described over the past six months.

The use of technology was controlled through the following question: ‘How many hours do you use the internet per day?’, (response option being 1 = ‘Less than 1 h’, 2 = ‘Between 1–2 h’, 3 = ‘Between 3–4 h’, 4 = ‘More than 4 h’).

We used the Cyberbullying Questionnaires CCB_A (aggressor) and CCB_V (victim) (Calvete et al., 2010), scoring 0–32 and 0–20, respectively, to evaluate cyberbullying. The Brief Symptom Inventory 18 (BSI-18) (Canavarro et al., 2017) assessed psychological distress through 18 items across three subscales: Somatization, Depression, and Anxiety. After finalizing the scale, a pretest confirmed all questions remained intact. The instrument was originally developed in Portuguese for the target population. As such, no translation or back-translation procedures were necessary. The instrument also included the BSI (Canavarro et al., 2017; Derogatis, 1993), a 53-item self-report questionnaire that evaluates psychological distress across nine symptom dimensions (e.g., depression, anxiety, somatization). Responses are given on a 5-point Likert scale ranging from 0 (‘not at all’) to 4 (‘extremely’). The BSI has shown robust psychometric properties, with Cronbach’s alphas in somatization (α = 0.874), depression (α = 0.821) and anxiety (α = 0.867). In the present study, the BSI was used to estimate discriminant validity.

### 2.1. Procedure

The schools from the focus groups participated in this study. All 6th-grade and higher students were invited via an email sent to schools detailing the study’s aims. Headmasters approved the study, and parents provided consent. Students then confirmed their participation and consented before completing the online questionnaire in class with teachers and investigators available for support. Participation was voluntary, anonymous, and approved by the Ministry of Education (0789800001) and by the University’s Ethics Committee that hosted the study (3/2021).

**Table 1:** List of items of the instrument.

Victimization items of Cyber Interpersonal Violence
I received insulting messages, photos or images They impersonated me and used my social media I received unwanted sexual images/messages and/or requests via email/message/social media ** They posted private content about me without permission ** They accessed my social media or email without permission They recorded a video or took photos of me while someone made fun of me or hurt me ** Posted and/or shared photos of me naked or with little clothing on the internet * I was pressured to talk about sex online * They spread rumours or false things about me They used my passwords (phone, social networks, email) to check my accounts without permission
Perpetration items of Cyber Interpersonal Violence
I sent insulting messages, photos or images I pretended to be someone else and used their social media I sent unwanted sexual images/messages and/or requests via email/message/social media * I posted private content about someone else without permission Accessed someone else’s social media or email without permission I recorded a video or took photos of someone while they mocked and/or hurt another person Posted and/or shared photos of someone undressed or with little clothing * I pressured someone to talk online about sex * Spread rumours or false things about someone else Used someone else’s passwords (phone, social media, email) to view their accounts without permission *
Bystander’s perpetration items of Cyber Interpersonal Violence
I know and/or have seen someone send insulting messages, photos or images I know and/or watched someone impersonate someone else while using their social media I know and/or watched someone send unwanted sexual images/messages and/or requests via email/text/social media I know and/or watched someone post private content about other people without permission ** I know and/or watched someone access someone else’s social media email without permission ** I know and/or watched someone recording a video or taking photos while mocking and/or hurting another person I know and/or saw someone posting and/or sharing photos of a person undressed or wearing little clothing I know and/or watched someone pressure another person to talk online about sex ** I know and/or have watched someone spread rumours or false things about a person ** I know and/or watched someone use someone else’s passwords (phone, social media, email) to check their accounts without permission **
Bystanders’ victimization items of Cyber Interpersonal Violence
I know and/or have seen someone receive insulting messages, photos or images I know and/or watched someone pretend to be that person and use their social media I know and/or watched someone receive unwanted sexual images/messages and/or requests via email/text/social media I know and/or watched someone who posted private content without permission ** I know and/or have seen someone who has accessed social media or email without permission ** I know and/or watched someone they took a video of or took photos of while mocking the person or hurting them I know and/or watched someone who has posted and/or shared nude or scantily clad photos of themselves I know and/or watched someone who was pressured to talk about sex online ** I know and/or watched someone about whom rumours, or false things have spread ** I know and/or have helped someone who used passwords (phone, social media, email) to verify their accounts without permission

Note: * Items eliminated due to low frequency; ** Items eliminated during exploratory analysis.

### 2.2. Data Analysis

The validation process of the scale began with an Exploratory Factor Analysis (EFA), conducted using the original ordinal-level data derived from a 5-point Likert scale, in accordance with the methodological assumptions of factor analysis. The CyIVIA was divided into two distinct sections: the first addressed victimization and perpetration factors, while the second focused on bystanders’ experiences with both victimization and perpetration. EFA was conducted using varimax rotation, and components with eigenvalues greater than 1 were retained. Items with factor loadings equal to or greater than 0.40 were considered to load adequately onto the corresponding factor. To evaluate internal consistency, Cronbach’s alpha coefficients were calculated for each retained factor. In a subsequent analysis aimed at estimating prevalence, variables were dichotomized, with responses recoded as 0 (never) and 1 (occurred at least once in the past six months). Items with an endorsement rate below 3% across all dimensions were excluded due to low representativeness.

Confirmatory factor analysis (CFA) was used to test how well the items represented the structure of the two different sections of CyIVIA. Maximum Likelihood Estimation (MLE) was utilized. Model fit was assessed through various descriptive criteria: the Comparative Fit Index (CFI), which measures how well the model improves upon a baseline model assuming uncorrelated observed items; Normed Fit Index (NFI) used to assess the goodness of fit of a proposed model compared to a null model; The Goodness-of-Fit Index (GFI) to assess the proportion of variance and covariance in the data that the model explains; the Standardized Root Mean Square Residual (SRMR); and the Root Mean Square Error of Approximation (RMSEA) with its 90% confidence interval (RMSEA 90% CI). Following Brown’s recommendations (Brown, 2015), model fit was classified as “adequate” if RMSEA and SRMR values were below 0.08 and CFI; NFI and GFI exceeded 0.9. A “good” fit was indicated by RMSEA and SRMR values below 0.05, CFI, NFI and GFI above 0.95. Values of SRMR < 0.08 are generally considered an acceptable fit. Factor loadings were also examined, with values of 0.3 or higher considered indicative of a variable’s relevance to the measured construct in each domain. CFA was conducted using EQS 6.1 software (Bentler, 1995).

Descriptive statistics were first computed to characterize the sample and summarize the main variables. As the assumption of normality was violated, non-parametric statistical tests were subsequently applied. Spearman’s correlations were used to explore associations between variables. Group comparisons were conducted using the Mann–Whitney U test for two-group comparisons and the Kruskal–Wallis test for comparisons involving more than two groups. Differences in prevalence rates by gender were examined using the chi-square test.

All statistical analysis was performed using IBM^®^ SPSS^®^ 29 for Windows, with a significance level of 0.05.

## 3. Results

### 3.1. Exploratory Factor Analysis

The results of the exploratory factor analysis model are presented in the tables below, based on the structure of two sections of CyVIA: victimization and perpetration factors and bystanders’ experiences with victimization and perpetration. In both analyses, the principal components method was applied, with the extraction of factors with eigenvalues above 1.0 and factor rotation using the Varimax method with Kaiser normalization (*KMO* = 0.824 vs. 0.746, respectively). Items with low communalities and undefined loading factors were eliminated from the analysis. Saturation with values greater than 0.40 are shown and the items are sorted by factor and magnitude for easier reading. For each section, 2 factors emerge with eigenvalues greater than 1.00 and explaining a total variance of 60.98% and 57.66%, respectively.

The results in Table 2 show that factor 1 has an eigenvalue of 4.898 and a variance percentage of 44.52. This factor corresponds to the perpetration scale with the original items showing saturations between 0.864 and 0.509. Factor 2 has an eigenvalue of 1.810 and a variance percentage of 16.46. This factor corresponds to the victimization scale. The original items showed saturations between 0.801 e 0.578.

The results in Table 3 show that factor 1 has an eigenvalue of 5.036 and a variance percentage of 45.78. This factor corresponds to the bystander perpetration scale with the original items showing saturations between 0.865 and 0.596. Factor 2 has an eigenvalue of 1.307 and a variance percentage of 11.88. This factor corresponds to the scale bystander victimization. The original items showed saturations between 0.739 and 0.535.

#### 3.1.1. Reliability

The descriptive statistics, Spearman correlation, and reliability analysis of the CyIVIA victimization and perpetration factors, and bystanders’ experiences with victimization and perpetration are present in Table 4 and Table 5.

Considering the values presented for Cronbach Alpha and the criteria proposed in the literature, according to which reliability is appropriate when Cronbach’s α is preferably above 0.80 (Kaplan & Saccuzzo, 2001), the four CyIVIA factors are consistent and consequently reliable in assessing victimization, perpetration and bystanders’ performance in CyIVIA.

Convergent validity was assessed using Spearman correlations, providing convergent evidence for the instrument’s validity. While some items, such as 3.6 (r = 0.468) and 4.6 (r = 0.449), showed moderate correlations with the global index (IGG), others, like 3.1 (r = 0.130) and 3.3 (r = 0.166), demonstrated weaker contributions. Inter-item correlations ranged from 0.125 to 0.640, indicating that items measured related but distinct aspects without redundancy. Similar analyses for CCB_A and CCB_V supported these findings, with moderate to strong correlations for items such as 1.9 (r = 0.589) and 1.6 (r = 0.445), alongside weaker correlations (e.g., 3.5, r = 0.106). Some high inter-item correlations, such as between 1.5 and 1.6 (r = 0.845), suggest potential redundancy. These results validate the instrument while highlighting the need to refine weaker items and reduce redundancies for greater robustness.

#### 3.1.2. Confirmatory Factor Analysis

**Figure 1:**
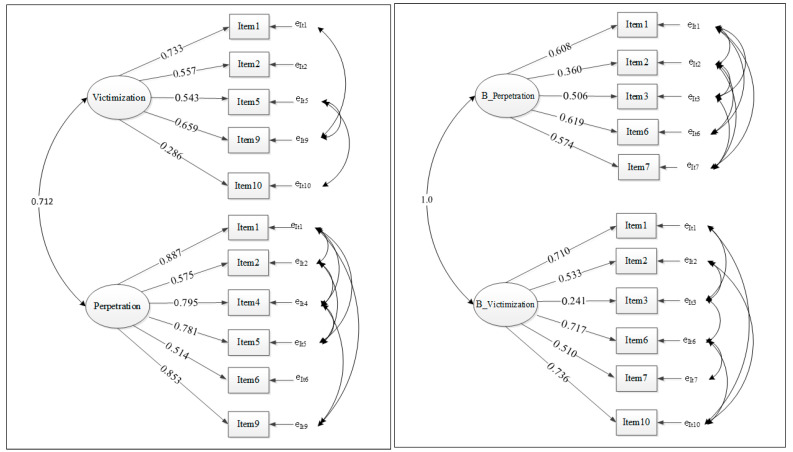
Factor loadings of confirmatory factor analysis for CyIVIA victimization and perpetration factors and for CyIVIA bystanders’ victimization and perpetration factors, respectively. Caption: Victimization: Item 1 = 0.733; item 2 = 0.557; item 5 = 0.543; item 9 = 0.659 and item 10 = 0.286. Perpetration: Item 1 = 0.887; item 2 = 0.575; item 4 = 0.795; item 5 = 0.781; item 6 = 0.514 and item 9 = 0.853. Bystanders_perpetration: Item 1 = 0.608; item 2 = 0.360; item 3 = 0.506; item 6 = 0.619 and item 7 = 0.574. Bystanders_victimization: Item 1 = 0.710; item 2 = 0.533; item 3 = 0.241; item 6 = 0.717; item 7 = 0.510 and item 10 = 0.736. **Note:** Single-headed arrows represent the factor loadings of each item on its respective latent variable (Victimization or Perpetration for direct involvement; Bystanders_Victimization or Bystanders_Perpetration for bystander involvement). Double-headed curved arrows represent correlations between latent variables and between item residuals (error terms). All standardized loadings are statistically significant (*p* < 0.001).

The multidimensional CyIVIA model is represented in Figure 1. Each model consists of two correlated factors: one addressing victimization and perpetration directly (Section 1 or indirectly, as bystander also in both ways.

The results of the CFA on the Portuguese version of CyIVIA—for victimization and perpetration factors, revealed that the 10-item, 2-factor model of the CyIVIA (latent variables: victimization and perpetration) provided an acceptable fit for the data. All items significantly loaded their hypothesized factors with acceptable fit indices: NFI = 0.908, CFI = 0.926, GFI = 0.918, SRMR = 0.064 (Arbuckle, 2011; Marôco, 2014) and RMSEA = 0.117, 90% IC [0.097, 0.137].

The results of the CFA on the Portuguese version of CyIVIA—bystanders’ experiences with victimization and perpetration, revealed that the 10-item, 2-factor model of the CyIVIA (latent variables: bystanders’ victimization and perpetration) provided an acceptable fit for the data. All items significantly loaded their hypothesized factors with acceptable fit indices: NFI = 0.908, CFI = 0.926, GFI = 0.918, SRMR = 0.064 (Arbuckle, 2011; Marôco, 2014) and RMSEA = 0.117, 90% IC [0.097, 0.137].

#### 3.1.3. Prevalence of Cyber Interpersonal Violence

Among the sample, 56.5% experienced CIV as victims, aggressors, or bystanders, with 44.7% reporting victimization. Common behaviors included spreading rumors (33.6%), unauthorized social media access (15.4%), and receiving insults (13%) (Table 6).

Regarding CIV perpetration, 17.8% of adolescents admitted involvement. The most frequent behaviors included sending insults (8.7%), spreading rumors (7.5%), and recording videos or photos mocking others (4.7%) (Table 7).

At least 46.2% of adolescents reported being bystanders, with 33.2% witnessing victimization and 42.3% observing aggression. Frequent behaviours included spreading rumours (24.9%), impersonation (16.6%), insults (13%), and mocking via videos or photos (11.1%) (Table 8).

The most common bystander-observed victimization behaviors were spreading rumors (19.4%), receiving insults (14.6%), and mocking through videos or photos (8.3%) (Table 9).

#### 3.1.4. Variables Associated with CIV

The total of CyIVIA score was calculated by adding the responses of each section. To obtain the score for the four sections, we should add the values (0–5) obtained in the items that comprise each section. Section 1 (direct CIV): Victimization: items 1, 2, 5, 9 and 10; Perpetration: items 1, 2, 4, 5, 6 and 9. In Section 2 indirect CIV): Bystanders perpetration: items 1, 2, 3, 6 and 7; Bystanders victimization: items 1, 2, 3, 6, 7 and 10).

Gender differences were found in CyIVIA dimensions, using the Mann–Whitney test. The results present in Table 10 revealed statistically significant differences in victimization (U = 6265.0; *p* = 0.003) and perpetration (U = 6082.0; *p* < 0.001), indicating that women are more victimized than men (mean = 6.64 vs. mean = 6.16), and has higher perpetration scores (mean = 7.65 vs. mean = 7.26). However, for bystander roles, no significant gender differences were found. In bystander victimization (U = 7631.0 *p* = 0.691) and bystander perpetration (U = 7007.5, *p* = 0.064). These findings underscore girls’ vulnerability as victims and consistent bystanders, alongside boys’ greater propensity for extreme perpetration, emphasizing the need for tailored, gender-specific interventions.

The analysis revealed significant differences in cyber victimization (H = 29.96, *p* < 0.0019, perpetration (H = 30.15, *p* < 0.001), and bystander victimization (H = 39.20, *p* < 0.001), and bystander perpetration (H = 8.04, *p* = 0.045) across school years using the Kruskal–Wallis test (Table 11). Using the Bonferroni test for pairwise comparison results showed that eighth-grade students had the highest mean for victimization compared to 6th grade (7.19 vs. 5.93; *p* = 0.002), 7th grade (7.19 vs. 5.80; *p* < 0.01) and 9th grade (7.19 vs. 5.86; *p* = 0.016). Eighth-grade students also showed the highest mean for perpetration compared to the 6th grade (8.56 vs. 6.96; *p* = 0.013) and 7th grade (8.56 vs. 6.51; *p* < 0.001). Sixth-grade students also showed higher levels of perpetration than 7th grade students (6.96 vs. 6.51; *p* = 0.019). These findings suggest that cyber victimization and perpetration peak during the 8th grade, highlighting a critical period for targeted interventions to address and mitigate CIV among adolescents.

#### 3.1.5. Anxiety, Depression, Somatization and CyIVIA

CyVIA dimensions correlated positively with BSI scores, so the higher the victim’s exposure, the higher the anxiety, depression, and somatization symptoms. Related to perpetrators, as the perpetration exposure increases, the higher the somatization, followed by depression and anxiety. Bystanders had lower but still positive relationships (Table 12).

## 4. Discussion

This study developed and validated a new measure to assess adolescent CIV across victimization, perpetration, and bystander roles, addressing the lack of validated instruments for this population in Portugal.

The findings confirm the reliability of CyIVIA, with Cronbach’s alpha coefficients ranging from 0.743 to 0.851, aligning with the internal consistency standards observed in previous instruments, such as the European Cyberbullying Intervention Project Questionnaire (ECIP-Q), which also demonstrated coefficients above 0.80 (Espino et al., 2022). Factorial analysis revealed a clear, multidimensional structure, validating the proposed dimensions of CyIVIA. However, some lower factor loadings suggest the need to refine certain items. This highlights the complexity of capturing multifaceted phenomena like CIV, particularly in rapidly evolving cultural and digital contexts.

The prevalence of involvement in CIV observed in this study aligns with findings from international investigations. For instance, 56.5% of adolescents reported involvement as victims, perpetrators, or bystanders. This is comparable to Machado et al. (2024), who identified cyber victimization rates exceeding 60% among Portuguese adolescents (Machado et al., 2024). Similarly, Espino et al. (2022) reported high rates of victimization in cyberbullying (15.2%) and cyber dating violence (49.6%), reinforcing the relevance of the digital context as a domain for potentially harmful interactions (Espino et al., 2022). The gender dynamics in CIV behaviours were also analysed. In this study, girls reported higher victimization (M = 1.31) and bystander involvement (M = 2.35), while boys exhibited higher levels of perpetration (M = 0.38). These findings are consistent with previous literature (Espino et al., 2022; Hldalgo-Rasmussen et al., 2020; Machado et al., 2022, 2024; Novo et al., 2014; Pereira et al., 2016; Sánchez Jiménez et al., 2023; Winarti & Saudah, 2023; Zumalde et al., 2021), which found that girls were more frequently victims of cyberbullying and cyberdating violence. At the same time, boys were the primary perpetrators of traditional bullying and sexual harassment. However, it is worth reflecting that some gender differences may relate to how boys and girls report and live the experiences of violence, warranting further investigation (Rodríguez-deArriba et al., 2024). Grade-level analysis revealed heightened vulnerability among 8th-grade students, with higher rates of victimization (M = 1.76) and perpetration (M = 0.68). Espino et al. (2022) and Machado et al. (2024) also highlighted peaks in cyberbullying incidence during early adolescence (11–14 years), suggesting that targeted intervention programs for this age group could mitigate risks.

Adolescents’ qualitative perspectives from focus groups (Machado et al., 2024) further contextualize these findings by highlighting multiple forms and typologies of CIV, such as cyberbullying, cyber dating abuse, and cyberstalking, together with risk factors spanning individual (e.g., low self-esteem, impulsivity), relational (e.g., jealousy, revenge), and family levels (e.g., domestic violence, economic hardship). Participants also emphasized gendered vulnerabilities, describing girls as more frequently targeted by appearance-based aggressions and boys as less affected by insults. Notably, bystanders emerged as pivotal actors, either supporting victims or minimizing incidents, underscoring the ecological complexity of CIV and reinforcing the need for culturally and gender-sensitive interventions.

Another significant point is the role of bystanders. This study revealed that 46.2% of adolescents had found themselves in this position, frequently observing rumours (24.9%) and online insults (13%). The bystander intervention approach advocated by Machado et al. (2024) emerges as a promising strategy to empower young people to intervene in abusive situations, fostering a safer digital environment.

The findings align with prior research on the psychological impacts of cyber violence. Li et al. (2024) linked bullying and cyberbullying to adolescent mental health issues, emphasizing the need for comprehensive interventions. Our results also show positive connectivity between being victims and suffering from anxiety, while being a perpetrator connects with higher somatization levels. Jun (2020) highlighted power dynamics and anonymity as key factors in cyberbullying, with Winarti and Saudah (2023) noting the fluid roles of adolescents as both victims and perpetrators, reflecting blurred boundaries in digital interactions.

### Study Limitations

This study has limitations, including a small, convenience-based sample, reducing representativeness and generalizability. A larger sample could support subgroup analysis, exploratory factor analysis, and gender invariance testing, which are key in CIV research. As Machado et al. (2024) suggested, future studies should use larger samples and in-depth qualitative methods to capture adolescents’ perspectives better, considering their unique characteristics, such as immaturity and limited disclosure on sensitive topics like sex.

Another limitation lies in the CFA results, particularly the elevated RMSEA value, which indicates a suboptimal model fit. While the internal consistency and factorial structure of the instrument were acceptable, some factor loadings were marginal, suggesting the need for refinement of specific items. Future research should validate the scale in larger and more diverse samples to enhance the generalizability of findings and allow for gender and age invariance testing, improving the scale’s psychometric robustness. Due to the relatively small subgroup sizes by gender (n = 146 and n = 107), it was not possible to conduct a formal measurement invariance analysis. The separate analyses by group were exploratory and do not support conclusions about structural differences. Future studies with larger and more balanced samples should test model invariance to ensure the cross-group validity of the instrument.

This study has a limitation related to the fact that the same sample (N = 253) was used for both the EFA and CFA due to the restricted sample size. This may increase the risk of overfitting and inflate model fit indices. Future studies should aim to replicate the factorial structure using independent samples.

Lastly, this study did not allow covariations between items across different factors in the CFA. While this approach ensured a parsimonious factorial structure, it underestimated potential associations between victimisation and perpetration items. Future studies should further explore theoretically justified covariances to improve model fit. As so, future studies should use larger samples, repeat the confirmatory analyses, and test the questionnaire in diverse populations to assess its cultural applicability.

In conclusion, despite its limitations, CyIVIA is a valid tool for assessing CIV. Evidence-based interventions, which combine parental education, school programs, and digital regulations, are crucial to mitigating CIV. Considering victims’ and perpetrators’ perspectives and fostering digital skills (De Coninck et al., 2023) is essential for safer, inclusive online environments.

## 5. Conclusions

This study provides preliminary empirical support for the CyIVIA instrument as a valid and reliable measure of CIV among adolescents, addressing victimisation, perpetration, and bystander roles. The factorial structure was consistent with theoretical expectations, and internal consistency indices met psychometric standards, though some items may benefit from further refinement.

The high prevalence of CIV observed, particularly among 8th grade students, underscores the need for early, developmentally targeted interventions. Gender-based differences followed previously reported patterns, with girls reporting more victimization and boys more frequent perpetration. The positive correlations between CyIVIA scores and psychological distress indicators, such as anxiety and somatization, reinforce the mental health implications of digital violence.

While the findings are promising, they must be interpreted cautiously given the limited and non-representative sample. Future research should focus on confirmatory testing with larger and more diverse populations and consider integrating qualitative methods to understand adolescents’ experiences and perceptions better. Overall, CyIVIA represents a valuable contribution to the assessment and prevention of CIV in both research and applied settings.

## Figures and Tables

**Table 2:** Exploratory factor analysis for victimization and perpetration factors of CyVIA.

	Factor 1	Factor 2
2.1. I sent insulting messages, photos or images	0.689	
2.2. I pretended to be someone else and used their social media	0.828	
2.4. I posted private content about someone else without permission	0.857	
2.5. Accessed someone else’s social media or email without permission	0.864	
2.6. I recorded a video or took photos of someone while they mocked and/or hurt another person	0.509	
2.9. Spread rumours or false things about someone else	0.860	
1.1. I received insulting messages, photos or images		0.578
1.2. They impersonated me and used my social media		0.589
1.5. They accessed my social media or email without permission		0.801
1.9. They spread rumours or false things about me		0.623
1.10. They used my passwords (phone, social networks, email) to check my accounts without permission		0.784
Eigenvalues		1.810
% of Variance	44.52	16.46

**Table 3:** Exploratory factor analysis for bystanders’ experiences with victimization and perpetration of CyVIA.

	Factor 1	Factor 2
3.1. I know and/or have seen someone send insulting messages, photos or images	0.798	
3.2. I know and/or watched someone impersonate someone else while using their social media	0.865	
3.3. I know and/or watched someone send unwanted sexual images/messages and/or requests via email/text/social media	0.745	
3.6. I know and/or watched someone recording a video or taking photos while mocking and/or hurting another person	0.596	
3.7. I know and/or saw someone posting and/or sharing photos of a person undressed or wearing little clothing	0.770	
4.1. I know and/or have seen someone receive insulting messages, photos or images		0.739
4.2. I know and/or watched someone pretend to be that person and use their social media		0.704
4.3. I know and/or watched someone receive unwanted sexual images/messages and/or requests via email/text/social media		0.685
4.6. I know and/or watched someone they took a video of or took photos of while mocking the person or hurting them		0.535
4.7. I know and/or watched someone who has posted and/or shared nude or scantily clad photos of themselves		0.567
4.10. I know and/or have helped someone who used passwords (phone, social media, email) to verify their accounts without permission		0.634
Eigenvalues	5.036	1.307
% of Variance	45.78	11.88

**Table 4:** Descriptive statistics and Spearman Correlation coefficients: victimization and perpetration factors.

	Minimum	Maximum	Mean ± SD	Cronbach Alpha	1	2
Perpetration	6.00	22.00	7.42 + 2.68	0.851	1.000	0.629 **
Victimization	5.00	16.00	6.35 + 2.30	0.712		1.000

** *p* < 0.01.

**Table 5:** Descriptive statistics and Spearman Correlation coefficients: bystanders’ experiences with victimization and perpetration factors.

	Minimum	Maximum	Mean ± SD	Cronbach Alpha	1	2
Byst_Victimization	5.00	19.00	5.90 ± 2.23	0.827	1.000	0.633 **
Byst_Perpetration	6.00	20.00	6.66 ± 1.75	0.728		1.000

** *p* < 0.01.

**Table 6:** Prevalence rates for items (victimization).

	It Happened at Least on Time in the Last Six Months	Gender			
		Male	Female	χ^2^	*p*
1. I received insulting messages, photos or images	13%	48.5%	51.5%	1.323	0.25
2. They impersonated me and used my social media	9.9%	68.0%	32.0%	1.204	0.272
3. I received unwanted sexual images/messages and/or requests via email/message/social media **	9.9%	32.0%	68.0%	7.512	0.006
4. They posted private content about me without permission **	9.9%	32.0%	68.0%	7.512	0.006
5. They accessed my social media or email without permission	15.4%	61.5%	38.5%	0.277	0.599
6. They recorded a video or took photos of me while someone made fun of me or hurt me **	6.3%	62.5%	37.5%	0.161	0.688
7. Posted and/or shared photos of me naked or with little clothing on the internet *	0.8%	100%	0%	1.477	0.224
8. I was pressured to talk about sex online *	1.6%	100%	0%	2.979	0.084
9. They spread rumours or false things about me	33.6%	40.0%	60.0%	16.446	<0.001
10. They used my passwords (phone, social networks, email) to check my accounts without permission	6.7%	47.1%	52.9%	0.847	0.357

Note: * Items eliminated due to low frequency; ** Items eliminated during exploratory analysis.

**Table 7:** Prevalence rates for items (perpetration).

	It Happened at Least on Time in the Last Six Months	Gender			
		Male	Female	χ^2^	*p*
1. I sent insulting messages, photos or images	8.7%	81.8%	18.2%	5.739	0.017
2. I pretended to be someone else and used their social media	3.2%	50.0%	50.0%	0.201	0.654
3. I sent unwanted sexual images/messages and/or requests via email/message/social media *	0.8%	100%	0%	1.477	0.224
4. I posted private content about someone else without permission	2.4%	33.3%	66.7%	1.496	0.221
5. Accessed someone else’s social media or email without permission	3.2%	50.0%	50.0%	0.201	0.654
6. I recorded a video or took photos of someone while they mocked and/or hurt another person	4.7%	100%	0%	9.232	0.002
7. Posted and/or shared photos of someone undressed or with little clothing *	0.8%	100%	0%	1.477	0.224
8. I pressured someone to talk online about sex *	0%	0%	0%	0%	0%
9. Spread rumours or false things about someone else	7.5%	47.4%	52.6%	0.900	0.343
10. Used someone else’s passwords (phone, social media, email) to view their accounts without permission *	0.8%	100%	0%	1.477	0.224

Note: * Items eliminated due to low frequency.

**Table 8:** Prevalence rates for items (Bystanders perpetration).

	It Happened at Least on Time in the Last Six Months	Gender			
		Male	Female	χ^2^	*p*
1. I know and/or have seen someone send insulting messages, photos or images	13%	66.7%	33.3%	1.248	0.264
2. I know and/or watched someone impersonate someone else while using their social media	16.6%	50.0%	50.0%	1.226	0.268
3. I know and/or watched someone send unwanted sexual images/messages and/or requests via email/text/social media	7.5%	52.6%	47.4%	0.217	0.641
4. I know and/or watched someone post private content about other people without permission **	10.3%	34.6%	65.4%	6.331	0.012
5. I know and/or watched someone access someone else’s social media email without permission **	5.9%	33.3%	66.7%	3.881	0.049
6. I know and/or watched someone recording a video or taking photos while mocking and/or hurting another person	11.1%	57.1%	42.9%	0.004	0.949
7. I know and/or saw someone posting and/or sharing photos of a person undressed or wearing little clothing	6.7%	47.1%	52.9%	0.847	0.357
8. I know and/or watched someone pressure another person to talk online about sex **	4%	20.0%	80.0%	6.066	0.014
9. I know and/or have watched someone spread rumours or false things about a person **	24.9%	36.5%	63.5%	15.448	<0.001
10. I know and/or watched someone use someone else’s passwords (phone, social media, email) to check their accounts without permission **	4%	40.0%	60.0%	1.338	0.247

Note: ** Items eliminated during exploratory analysis.

**Table 9:** Prevalence rates for items (Bystanders victimization).

	It Happened at Least on Time in the Last Six Months	Gender			
		Male	Female	χ^2^	*p*
1. I know and/or have seen someone receive insulting messages, photos or images	14.6%	56.8%	43.2%	0.016	0.899
2. I know and/or watched someone pretend to be that person and use their social media	4.7%	50.0%	50.0%	0.307	0.580
3. I know and/or watched someone receive unwanted sexual images/messages and/or requests via email/text/social media	6.3%	50.0%	50.0%	0.416	0.519
4. I know and/or watched someone who posted private content without permission **	4.3%	18.2%	81.8%	7.361	0.007
5. I know and/or have seen someone who has accessed social media or email without permission **	5.1%	38.5%	61.5%	2.080	0.149
6. I know and/or watched someone they took a video of or took photos of while mocking the person or hurting them	8.3%	47.6%	52.4%	0.955	0.328
7. I know and/or watched someone who has posted and/or shared nude or scantily clad photos of themselves	6.3%	25.0%	75.0%	7.487	0.006
8. I know and/or watched someone who was pressured to talk about sex online **	3.2%	0%	100%	11.272	<0.001
9. I know and/or watched someone about whom rumours, or false things have spread **	19.4%	38.8%	61.2%	8.924	0.003
10. I know and/or have helped someone who used passwords (phone, social media, email) to verify their accounts without permission	0.8%	100%	0%	1.477	0.224

Note: ** Items eliminated during exploratory analysis.

**Table 10:** Results of the Mann–Whitney Test for Gender Comparison.

	Gender	N (%)	M (SD)	U	*p*
Victimization	Male	146 (57.7)	6.16 ± 2.35	6275.0	0.003
	Female	107 (42.3)	6.64 ± 2.22		
Perpetration	Male	146 (57.7)	7.26 ± 2.99	6082.0	<0.001
	Female	107 (42.3)	7.65 ± 2.16		
Byst_victimization	Male	146 (57.7)	5.92 ± 2.42	7631.0	0.691
	Female	107 (42.3)	5.86 ± 1.95		
Byst_perpetration	Male	146 (57.7)	6.62 ± 1.92	7007.5	0.064
	Female	107 (42.3)	6.73 ± 1.49		

**Table 11:** Results of the Kruskal–Wallis Test for Grade Comparison.

	Grade Level	N (%)	M (SD)	H (3)	*p*
Victimization	6th grade	107 (42.3)	5.93 ± 1.56	29.96	<0.001
	7th grade	41 (16.2)	5.80 ± 2.42		
	8th grade	91 (36.0)	7.19 ± 2.76		
	9th grade	14 (5.5)	5.86 ± 2.18		
Perpetration	6th grade	107 (42.3)	6.96 ± 1.47	30.15	<0.001
	7th grade	41 (16.2)	6.51 ± 1.85		
	8th grade	91 (36.0)	8.56 ± 3.72		
	9th grade	14 (5.5)	6.29 ± 0.47		
Byst_victimization	6th grade	107 (42.3)	5.24 ± 0.61	39.20	<0.001
	7th grade	41 (16.2)	5.73 ± 2.64		
	8th grade	91 (36.0)	6.81 ± 2.98		
	9th grade	14 (5.5)	5.43 ± 0.76		
Byst_perpetration	6th grade	107 (42.3)	6.45 ± 0.85	8.04	0.045
	7th grade	41 (16.2)	6.15 ± 0.53		
	8th grade	91 (36.0)	7.14 ± 2.63		
	9th grade	14 (5.5)	6.71 ± 1.44		

**Table 12:** Descriptive statistics and Spearman Correlation coefficients between CyIVIA and BSI.

	M ± SD	1	2	3	4	5	6	7
1. Victimization	6.36 ± 2.30	1						
2. Perpetration	7.43 ± 2.68	0.629 **	1					
3. Byst_victimization	5.90 ± 2.23	0.470 **	0.715 **	1				
4. Byst_perpetration	6.66 ± 1.75	0.390 **	0.615 **	0.534 **	1			
5. BSI_Somatization	9.57 ± 4.79	0.475 **	0.467 **	0.280 **	0.238 **	1		
6. BSI_Depression	8.90 ± 4.05	0.468 **	0.447 **	0.260 **	0.225 **	0.891 **	1	
7. BSI_Anxiety	9.09 ± 4.53	0.498 **	0.437 **	0.225 **	0.217 **	0.912 **	0.877 **	1

** *p* < 0.01.

## Data Availability

Anonymized data set will be available upon request to the corresponding author.

## Abbreviations

The following abbreviations are used in this manuscript:

BSI-18	Brief Symptom Inventory 18
CCB_A	Cyberbullying Questionnaires CCB_A (agressor)
CCB_V	Cyberbullying Questionnaires CCB_V (victim)
CDA	cyber dating abuse
CDAQ	Cyber Dating Abuse Questionnaire
CDVI	Cyber Dating Violence Inventory
CFA	Confirmatory factor analysis
CFI	Comparative Fit Index
CIV	cyber interpersonal violence
CYB-AGS	Adolescent Cyber-Aggressor Scale
CyIVIA	Cyber Interpersonal Violence Instrument for Adolescents
EFA	exploratory factor analysis
GFI	Goodness-of-Fit Index
NFI	Normed Fit Index
RMSEA	Root Mean Square Error of Approximation
SRMR	Standardized Root Mean Square Residual
